# Effects of virtual reality immersive training with computerized cognitive training on cognitive function and activities of daily living performance in patients with acute stage stroke: A preliminary randomized controlled trial: Retraction

**DOI:** 10.1097/MD.0000000000020598

**Published:** 2020-05-22

The article, “Effects of virtual reality immersive training with computerized cognitive training on cognitive function and activities of daily living performance in patients with acute stage stroke: A preliminary randomized controlled trial”,^[1]^ which appears in Volume 98, Issue 11 of *Medicine*, is being retracted by request of the authors and the developers due to a lack of a formal agreement between the authors of the article and the developers of the virtual reality program.
